# The time course and characteristics of procedural learning in schizophrenia patients and healthy individuals

**DOI:** 10.3389/fnhum.2015.00475

**Published:** 2015-09-01

**Authors:** Yael Adini, Yoram S. Bonneh, Seva Komm, Lisa Deutsch, David Israeli

**Affiliations:** ^1^The Institute for Vision ResearchKiron, Israel; ^2^Department of Human Biology, University of HaifaHaifa, Israel; ^3^Department of Optometry, Mina and Everard Goodman Faculty of Life Sciences, Bar-Ilan UniversityRamat-Gan, Israel; ^4^Day Care Unit and the Laboratory of Imaging and Brain Stimulation, Kfar Shaul hospital, Jerusalem Center for Mental HealthJerusalem, Israel; ^5^Biostats Statistical Consulting LtdModiin, Israel; ^6^Jerusalem Center for Mental Health, Hebrew UniversityJerusalem, Israel

**Keywords:** serial reaction-time task, procedural learning, skill learning, motor learning, sequence learning, statistical learning, chunking, schizophrenia

## Abstract

Patients with schizophrenia have deficits in some types of procedural learning. Several mechanisms contribute to this learning in healthy individuals, including statistical and sequence-learning. To find preserved and impaired learning mechanisms in schizophrenia, we studied the time course and characteristics of implicitly introduced sequence-learning (SRT task) in 15 schizophrenia patients (seven mild and eight severe) and nine healthy controls, in short sessions over multiple days (5–22). The data show speed gains of similar magnitude for all groups, but the groups differed in overall speed and in the characteristics of the learning. By analyzing the data according to its spatial-position and temporal-order components, we provide evidence for two types of learning that could differentiate the groups: while the learning of the slower, severe group was dominated by statistical learning, the control group moved from a fast learning phase of statistical-related performance to subsequence learning (chunking). Our findings oppose the naïve assumption that a similar gain of speed reflects a similar learning process; they indicate that the slower performance reflects the activation of a different motor plan than does the faster performance; and demonstrate that statistical learning and subsequence learning are two successive stages in implicit sequence learning, with chunks inferred from prior statistical computations. Our results indicate that statistical learning is intact in patients with schizophrenia, but is slower to develop in the severe patients. We suggest that this slow learning rate and the associated slow performance contribute to their deficit in developing sequence-specific learning by setting a temporal constraint on developing higher order associations.

## Introduction

Procedures, skills, and tacit knowledge are involved in almost every aspect of our everyday activities: from perception and action to language, social behavior, and problem solving. Skillful performance evolves through practice, and is implied by improved performance relative to the baseline (Karni and Sagi, 1993; Karni et al., 1998; Censor et al., 2012; Shmuelof et al., 2012). It can be acquired as well as assessed by non-declarative means (Nissen and Bullemer, 1987; Reber, 1993; Seger, 1994; Squire, 1994), and is the subject of extensive research in varied human populations. One of these populations is patients with schizophrenia, which are known to be impaired in performing daily procedures, and have deficits in specific types of procedural learning, such as sequence-learning measured with the serial reaction time (SRT) task. Several learning mechanisms contribute to implicit procedural learning in healthy individuals, including statistical and subsequence-learning (chunking). To find preserved and impaired mechanisms of procedural learning in schizophrenia, we studied the evolution in time and the characteristics of implicitly introduced sequence learning (SEQL) in patients and in normal controls. This approach allowed us to explore open research questions regarding procedural learning, and to identify preserved and impaired information processing mechanisms in schizophrenia.

### Skills, Procedures, and Sequences of Instructions

Perceptual and motor skill learning are implied by measures that include speed, error rate, fluency, and smoothness (e.g., Shmuelof et al., 2012). Skillful performance evolves in a non-linear rate, through distinctive phases that depend on two crucial factors: the amount of repetitions and the elapsed time, including the time between the practice sessions, sometimes in sleep (Karni and Sagi, 1993; Karni et al., 1995, 1998; Stickgold et al., 2000; Korman et al., 2003; Ofen-Noy et al., 2003; Walker et al., 2003; Press et al., 2005; Censor et al., 2012). Skillful motor performance was taken to imply the existence of an underlying “motor plan” at different abstraction levels (Clegg et al., 1998; Chafee and Ashe, 2007). In its advanced form, a motor procedure can be viewed as a fixed (but changeable) sequence of motor instructions (Hikosaka et al., 2002). Consequently, the terms procedural learning and implicit SEQL were sometimes used as tightly linked concepts (Willingham et al., 1989; Knopman and Nissen, 1991; Green et al., 1997).

### Sequence Learning

Sequence learning refers to human’s ability to link between external and/or internal temporal events, between elements in a stream of information, and/or a sequence of actions or movements. Sequences can be learned explicitly, according to the temporal order of the items’ presentation (serial order), in a conscious, declarative manner, e.g., as in the finger tapping paradigm (Korman et al., 2003). However, implicit SEQL is based on human’s ability to automatically extract commonalities, regularities, and transitional probabilities from a given set of repeating information. This ability stands at the basis of our very fundamental perceptual, motor, and cognitive abilities, including motor coordination, language acquisition, social cognition, planning, prediction, reasoning, intuition, and decision making (Reber, 1993; Saffran et al., 1996; Clegg et al., 1998; Lieberman, 2000; Page and Norris, 2009). SEQL can be acquired automatically, in the sense that it can occur without intention or awareness, in an incidental, non-episodic manner (Nissen and Bullemer, 1987; Reber, 1993; Seger, 1994; Squire, 1994). It depends on the amount of practice and the frequency of the repeated regularities (see Page and Norris, 2009, for a summary). This type of learning involves several brain mechanisms including simple associative brain mechanisms (i.e., Hebb and anti-Hebb rules; Nissen and Bullemer, 1987; Seger, 1994; Clegg et al., 1998; Curran, 1998) that allow for statistical learning (Stadler, 1992; Reed and Johnson, 1994; Saffran et al., 1996; Curran, 1998; Hunt and Aslin, 2001; Perruchet and Pacton, 2006) and information chunking processes (Gobet et al., 2001; Sakai et al., 2003) at multiple, probably hierarchical abstraction levels (Curran, 1995, 1998; Clegg et al., 1998; Graybiel, 1998; Koch and Hoffmann, 2000). The SRT task (Nissen and Bullemer, 1987) has proven to be a useful model to explore implicit SEQL and its underlying neuronal mechanisms for various human populations (e.g., Curran, 1995; Jimenez, 2008).

### The SRT Paradigm

**FIGURE 1:**
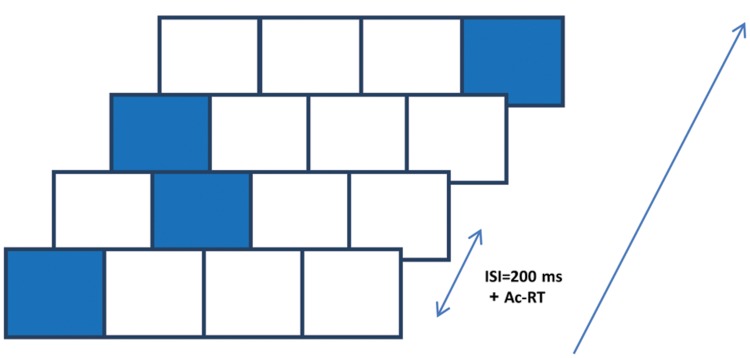
**The serial reaction time (SRT) paradigm implemented on a touch tablet.** Participants were presented with four squares (2 cm × 2 cm each, referred to as 1–4 from left to right) that were “lit up” in an apparently random sequence of 12 spatial positions, cyclically repeated 8 times (total of 96 presentation). They were instructed to touch the highlighted positions with their preferred index finger “as fast as possible,” upon which the next position was highlighted after 200 ms. The highlighting persisted on the screen until an accurate response was made.

The SRT paradigm is a four-choice reaction time task in which visual cues are linked to spatial-specific motor responses (Nissen and Bullemer, 1987; Robertson, 2007). In one of its forms, which we used here, visual cues appear in any one of four possible positions arranged horizontally on a touch tablet (see **Figure 1**); the responses are made by rapidly touching the cued location with a single finger. The cues are presented in a fixed, structured series of spatial locations; thus, unbeknown to the subjects, the cues introduce a sequence of lateral movements to be learned (Richard et al., 2009). In the classical SRT paradigm, there are two measures of learning: the reaction time for the complete series (RT), and the sequence-specific learning (RT for a trained sequence is faster than RT for a new, random, or pseudorandom sequence; Robertson, 2007). However, studies that explored the possibility that learning in the SRT task includes a statistical learning component used the pattern of RT differences that correlates with the underlying temporal order and/or the specific spatial positions as a dependent measure. This measure allows one to assess the learners’ ability to exploit different statistics embedded in the input, across time (Hunt and Aslin, 2001). To the best of our knowledge, this method has not been studied in schizophrenia, until now. In the current study we explored two components of SEQL: statistical learning and subsequence learning. These two components can be inferred from the spatial position and the serial order analyses, respectively (see below).

### SRT and Schizophrenia

The relative simplicity of the SRT paradigm, the possibility to introduce and learn the sequence properties implicitly, and the performance-based measure of learning make it a convenient paradigm for studying procedural learning in subjects with various psychiatric, neurological, and cognitive disorders, one of which is schizophrenia. Schizophrenia is a brain-based disease with yet unclear neuropathology (Harrison, 1999). It affects basic human functions such as perception, motor coordination, cognition, social interactions, language, and thought (Heinrichs and Zakzanis, 1998; Harrison, 1999). Since all of these functions are based, at least to some extent, on implicit learning, procedures, and tacit knowledge, many studies have sought to investigate whether procedural learning is intact in schizophrenia patients. Moreover, schizophrenia patients also suffer from difficulties in initiating actions, low motivation, and attention deficits, which typically lead to poor and fragmented performance in various skills. Since the acquisition of a new skill is generally associated with a decrease in the need for effortful control over performance, and the development of automaticity via consolidating task-specific procedures (Poldrack et al., 2005), the ability of the patients to master skills, if it exists, might be used to overcome or bypass their initiation and motivational deficits and thus, it has important therapeutic implications.

Green et al. (1997) explored procedural learning in schizophrenia via the SRT task. They found comparable RT improvement in both the patients and controls; however, the patients performed significantly slower than the controls and exhibited significantly less sequence-specific learning. Green et al. (1997) suggested that the sequence-unspecific learning may reflect an improvement in the pairing of visual cues and the corresponding finger-response (Green et al., 1997, see also Curran, 1995; Robertson, 2007). Following this work, several studies showed that schizophrenia individuals can improve performance on the SRT task, as revealed by faster RTs; however, the evidence regarding their ability to achieve sequence-specific learning is conflicting, with variations in task structure, the amount of practice, and the medication status of patients playing a role in the findings (Green et al., 1997; Perry et al., 2000; Marvel et al., 2007; Foerde et al., 2008; Siegert et al., 2008).

In a critical review of the literature on SEQL in schizophrenia, Remillard (2014) argued that the current evidence does not allow one to distinguish between deficits in the acquisition of sequence knowledge and the expression of this knowledge. He suggested that the reported deficit could arise from a deficit in expressing sequence knowledge via an anticipatory process, due to the slow disengagement of attention that characterizes schizophrenia patients. In support of the hypothesis of intact implicit knowledge acquisition, patients with schizophrenia were found to be capable of implicitly learn complex rule-based knowledge, as assessed by the artificial grammar paradigm, although they needed more practice than did healthy controls (Danion et al., 2001; Horan et al., 2008).

Current studies of SRT in schizophrenia have two limitations: (1) they are typically based on 5–10 practice blocks within a single training session, that cannot accommodate a possible slower learning rate in the patients, and (2) they focus on learning the specific sequence as the primary (or even the only) indicator of procedural learning (Green et al., 1997; Siegert et al., 2008). In addition, these studies excluded from the analysis some measurements that otherwise could reflect the rate of errors and the smoothness of performance (e.g., trials with very long reaction times), which are prominent measures in procedural learning. To resolve these potential problems, we used multiple practice sessions lasting many days, and analyzed procedural learning at different abstraction levels, looking for evidence of statistical learning, chunking learning, and procedural (skill) learning.

### Statistical-Related Learning in the Current Study

Statistical learning enables the subjects to increase their speed by a successful probability-based prediction of the next movement or some of its components. Successful prediction allows for the preparation and initiation of the next movement even before the next cue appears. In the current study, predictions of the lateral finger movement that is needed for rapidly touching the cued location (**Figure 1**) have two components: direction and size. As previously argued (Hunt and Aslin, 2001), a random sequence of visual cues does not allow for any position-specific improvement. However, the SRT sequence is typically designed, as in the current study, with an equal probability of transition between position pairs, and with no transition to the same position in consecutive trials. As a result, the direction of movement from the lateral positions is deterministic (to the right from the left-most position and to the left from the right-most position). Therefore, on average, it should allow for faster responses to the central positions. This is because the direction of motion is fully predictable for two out of the three possible transitions toward each central position (e.g., from p1 to p2; see **Figure 1**), and for only one out of the three possible transitions toward each lateral position. The predicted statistical-related learning could thus be assessed by analyzing the pattern of RT for the different spatial positions, and its relationship to the serial order in the sequence. A non-specific learning component should show same RT gains for all four spatial positions. Statistical-related learning should result in faster RTs for central compared with lateral positions, regardless of the serial positions in the sequence. When the learning of the specific sequence or parts of it occurs, the spatial position’s specific distinctions are expected to fade off.

### Subsequence Learning

Humans have the ability to implicitly divide a chain of information into chunks (subsequences). This ‘chunking’ process was found to be a key mechanism underlying information processing in perceptions, actions, learning, and cognition (Gobet et al., 2001). For example, acquiring a complex sequential skill involves chaining a number of elementary movements (“chunks”) to make a complete sequence. A chunk is defined as “a collection of elements having strong associations with one another, but weak associations with elements within other chunks” (Gobet et al., 2001). It serves as a memory unit, or in our case, a single motor plan. In the SRT paradigm, a chunking process can be inferred from serial order analysis of the RTs. In our analysis, we expect that when a subject learns a subsequence, elements that belong to the same chunk will be faster than the neighboring elements.

### The Current Study

There were three aims of the current research: (1) to measure the time course, the dynamic characteristics, and the rate of implicitly introduced SEQL, in patients with schizophrenia as compared to healthy controls, and (2) to identify and characterize impaired and preserved mechanisms in this cognitive-related visual-motor task (i.e., the acquisition of statistical knowledge and of serial-order knowledge). (3) To test the ability of schizophrenia patients with severe, predominantly negative symptoms to develop and retain new skills. The multi-session format of the study, which involved months of experimental recording, was chosen to overcome a potential limitation of the previous typical single-session SRT studies, which could have been affected by the slower learning rate of the patients.

## Materials and Methods

### Participants

A group of 15 patients participated in the study. The patients met DSM-IV criteria for schizophrenia and were recruited from a day hospitalization patients’ population from a public psychiatric hospital (**Kfar Shaul Mental Health Center**). Patients were screened for medical and neurological conditions. Subjects with Parkinson disease and/or drugs or alcohol addiction were excluded. Psychiatric symptomatology was assessed by the Positive and Negative Syndrome Scale (PANSS). The schizophrenia patients were clinically classified in correlation with the dominancy and severity of their negative symptoms and were divided into two equal groups: severe (SEV group, *N* = 8) with PANSS-negative ≥12 and mild (MILD group, *N* = 7) with PANSS-negative less than 12. The clinical and demographic data of the groups appear in **Table 1**. The PANSS-negative values were significantly different between the groups, *p* < 0.001. All other demographic and clinical parameters, including PANSS-positive, illness duration, age of onset and age, had a large overlap between groups and did not differ significantly (see **Table 1**). All schizophrenia patients received atypical neuroleptic medication in equivalent doses, (risperidone 2–3 mg, quetiapine 400–600 mg, olanzapine 10–15 mg, and clozapine 300–450 mg) and were stabilized on medication prior to entering the study. The control group comprised nine age-matched subjects, recruited from the hospital community.

**Table 1:** Demographic and clinical data of the participants.

	Control (CONT)	Mild (MILD)	Severe (SEV)
Age	33 ± 3.7	32.9 ± 4.1 (19–44)	35.2 ± 4.1 (23–48)
Gender	6F/3M	4F/3M	1F/7M
Age of onset		21.5 ± 3.2 (18–27)	25.8 ± 4.1 (23–33)
Illness duration (years)		11.4 ± 4.1 (0.45–25)	8.2 ± 4.1 (0.16–27)
PANSS Positive		10.9 ± 0.72 (8–13)	11.7 ± 3.1 (7–18)
PANSS Negative		9.1 ± 0.76 (7–11)	22 ± 3.01 (12–35)
Number of practice days^∗^	11.5 ± 2.9 (8–14)	9.1 ± 3.7 (5–14)	8.8 ± 4.4 (5–14)

*Positive and Negative Syndrome Scale (PANSS) denotes positive-and-negative symptoms scores in the range 7–49. The number of practice days was averaged per group, excluding sessions beyond 14 days. The numbers in each column correspond to the average value, ± *SD*, and the range of the individual data appear in parentheses.*

The study was carried out in accordance with the recommendations of the Helsinki committee of the Jerusalem mental health center affiliated with the Hebrew University with a written informed consent from all subjects.

### Apparatus and Procedure

Subjects were seated in front of a touch-sensitive computer screen and viewed a static horizontal template of four adjacent squares (black lines on a gray background), each having a 2 cm × 2 cm width. On each trial one square was highlighted in blue. The subjects were asked to touch the highlighted square (the visual cue) ‘as fast as possible.’ The visual cue stayed on the screen until the subject made contact with the screen at the highlighted location. The time for an accurate response (from the target onset until the subject touched the visual cue location) was recorded. A new visual cue was then presented after a response–stimulus interval (RSI) of 200 ms. Unbeknown to the subjects, the visual cue followed a repeating sequence of locations. Designating the spatial positions as 1–4 from left to right (**Figure 1**), we used four different sequences: in the main experiment the sequence (S12) was 1-2-1-4-3-2-4-1-3-4-2-3, as previously used (Jimenez and Vazquez, 2005). This sequence is a second-order-conditional (SOC) sequence, where each element has the same frequency and can be predicted only from the identity of at least two preceding elements (Curran, 1998). Successive trials were not allowed to appear at the same spatial position. One block of trials contained eight successive (cyclic) repetitions of the fixed 12-element sequence, making a total of 96 trials in a block. The subjects performed three successive blocks in a session, a single session for each training day, and the practice lasted 5–22 training days (see **Table 1** for the group average and range). On average, all groups practiced for a similar number of days, and no correlation was found between any of the parameters and the number of practice days, including age, age of illness onset, illness duration, as well as positive and negative symptoms (*p* > 0.17 under all conditions). Most patients trained for 14 training days or less, but one mild patient trained for 22 days and three severe patients trained for 16, 20, and 22 days,. We thus chose the 14th day as the “last day of training” for the group average data, and presented the full training data for selected individuals (**Figure 3**). The interval between the practice days was 5 (*SD* = 0.45, *N* = 15 patients) and 12.2 (*SD* = 2.2, *N* = 9 controls) days on average.

To determine whether the slow performance of the severe patients, even after extensive practice, depends on the sequence properties, we tested four severe patients, with a shorter, 7-element hybrid sequence (S7), which was ‘1-2-1-4-3-2-4’; note that this sequence is composed of the first seven elements of S12. These four patients practiced S12 for 10–22 days and reached a saturated performance, i.e., they did not improve for several practice days. Then they practiced S7 for three practice blocks per session. In this experiment we had 98 trials per block (14 repetitions × 7 elements).

### Transfer Tests

To determine the specificity of the learning to the training sequence (S12) after extensive training, we conducted a transfer test, in a subset of the patients. Only patients that underwent extensive training (equal or >8 days) were tested: four severe patients, but just one mild patient, and seven controls. The new sequence that we used was the 12-element SOC 3-2-4-1-3-1-2-3-4-2-1-4; it was denoted as R12. Tests for the transfer of learning were conducted on average after 13 practice days (39 blocks). Three control subjects, who displayed an additional improvement in speed after the first test for transfer (8 days on average), performed another test for transfer at a later practice stage (after 19 days on average). For each participant we computed: (1) RT of the first block of S12 (RT0), which served as the baseline measure to compute RT gains; (2) RT(S12) of the last practice block before the transfer test; (3) RT(R12) of the first succeeding block, for the new sequence R12. We then computed two measures for transfer: (a) The percentage of transfer, defined as [RT(R12)–RT0]/[RT(S12)–RT0]^∗^100, with 100% transfer implying non-specific learning; (b) Sequence-specific gain, defined as RT(S12)–RT(R12). Two of the four severe patients that practiced the 7-element sequence S7 were also tested with another 7-element sequence R7, which was 3-4-1-2-1-4-2.

### Statistical Analysis

Data analysis was performed using SAS^®^ v9.3 (SAS Institute, Cary, NC, USA). A *p*-value of 0.05 was considered statistically significant and nominal *p*-values are presented.

We compared the groups per day, using repeated measures analysis of variance (SAS PROC Mixed). At first the per-day (up to day 14) pairwise comparisons of the LSmean (model estimated adjusted means) differences in RT between groups were performed. Before fitting the model the 96 trials of each block within day were averaged; thus, we modeled the (mean) RT as a function of the group and day, and the day-by-group interaction term, the day was entered as a random effect where the blocks within day per subject were treated as the correlated (repeated) measure. The same method was used to compare the mean RT across participants in the time scale of blocks.

Using repeated measures analysis of variance (SAS PROC Mixed) we compared between the groups and within the groups per day by spatial position. Before fitting the model the 24 trials of each spatial position within block within day were averaged, thus we model the (mean) RT as a function of group, spatial position and day and the day by group by spatial position interaction term, day was entered as a random effect where the blocks within day per subject were treated as the repeated measure. The within day pair wise comparisons between the groups for each spatial position and for spatial positions within groups are presented with respective 95% confidence intervals.

Inter-subject, intra-block (i.e., “intra-subject”), and inter-block (within session) variability were assessed by fitting several (one per day for days 1, 3, and 10) random effects models with the SAS PROC MIXED procedure, where the RT was modeled with the subject, and block within subject entered as random effects.

The *SD* were estimated from the variance components, and the overall mean RT, adjusted for the block, was estimated from the intercept and its 95% confidence interval. *SD* and the coefficients of variations (CV) of the three measures are presented with their respective 95% confidence intervals. The CV is a unit less measure of relative variability that is comparable across different measures.

The confidence intervals of the inter-subject *SD* and the inter-block (within session) *SD* and CVs were calculated with bootstrap methodology using 10,000 simulated samples. The *SD* are compared via the confidence intervals; if the confidence intervals overlap, then we stated that there is no statistically significant difference between the two parameters.

## Results

In the following, we will analyze the characteristics and properties of learning in the SRT task. We start by analyzing the time course of learning and its characteristics, including inter- and intra-subject variability, as well as online (within session) vs. oﬄine learning effects. We then consider the possible components of this learning, divided into (1) statistical learning via spatial specific learning, (2) serial-order learning, via subsequence learning (chunking), and (3) increased smoothness in performance via exploring intra-subject RT variability.

### The Time Course of Learning

**FIGURE 2:**
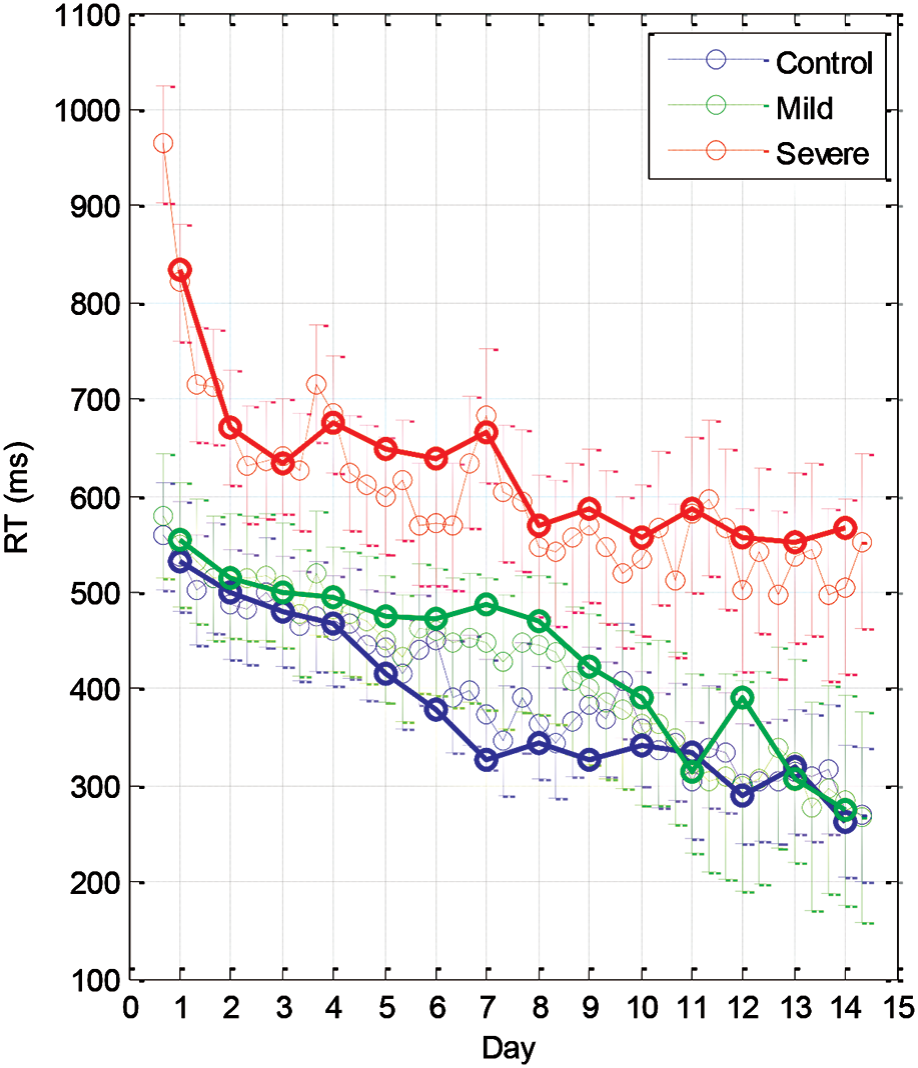
**Group learning curves.** The mean series RT across participants as a function of day (bold line) and block (three blocks per day, thin line) for the three experimental groups (SEV, *n* = 8, MILD, *n* = 7, CONT, *n* = 9) as estimated from the model (LSmeans, see Materials and Methods). The error bars denote the residual *SD*.

**FIGURE 3:**
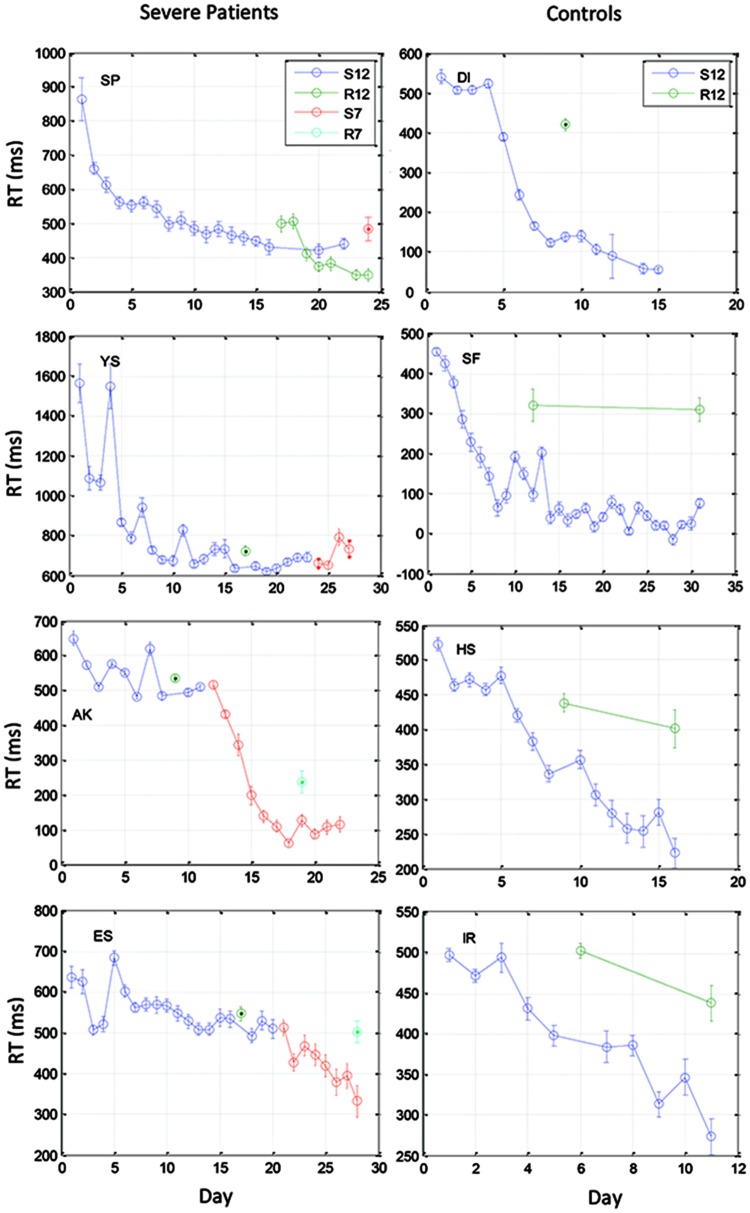
**Examples of learning curves for 4 severe patients **(Left)** and for four control subjects **(Right)**.** Data were averaged across three blocks of 96 trials on each training day. Data are shown for two 12-element sequences: S12 (the trained sequence) and R12 (a different 12-element sequence for testing transfer), and two 7-element sequences that were administered to the patients after they had reached a saturation level (S7 for training, R7 for test of transfer).

The learning-related improvement in the mean RT over blocks and practice days is shown in **Figure 2** (group averages) and **Figure 3** (individual examples). We first averaged the 96 trials per block, and then computed the LSmean (the model-estimated mean) RT across subjects, per block and per day (**Figure 2**). Using repeated measures analysis of variance (see the statistical analysis), we found statistically significant differences in the series mean RT between the SEV group and both other groups (MILD, CONT) for every single day. The CONT group was found to be similar to the MILD group (i.e., with no significant difference) on almost all days except days 6–8, where the CONT group became faster than the MILD group who, on average, started to “catch up” and improved their RT performance 4 days later (**Figure 2**).

We found that with training, the three groups exhibited a comparable gain of speed between the first and the last (14th) day of training. The average RT gains were 269, 288, and 267 ms for the CONT, MILD, and SEV groups, respectively. However, the pattern of learning differed among the groups, and showed opposite tendencies. The SEV group showed an initial short-term phase of fast learning (from day 1 to 3) in which the mean RT improved rapidly and accumulated to a total of ∼200 ms gain of speed relative to the first day of practice (75% of the total learning gain, **Figure 2**). This initial learning phase was followed by a long-term, slower learning stage in which additional performance gains were incrementally obtained over multiple sessions of training (one session per a practice day). This phase converged into a stable performance from the 11th day of practice. Two severe patients continued to practice up to 22 practice days (**Figure 3** subjects YS and SP), but they had no further speedup gains. In contrast, the other two groups exhibited a different pattern of learning, starting with a very slow rate of learning for the first 4 days (CONT) or even after 8 days of practice (MILD; see **Figure 3**, for individual examples), followed by a sudden increase in learning for 3 days, when it leveled off, and reached saturation.

The individual examples in **Figure 3** provide preliminary results regarding the ability of patients with severe schizophrenia to acquire performance speed which is comparable to normal. The figure shows that some, but not all of the severe patients improved their performance when they changed from a 12-element SOC sequence to a 7-element hybrid sequence.

### Online and Oﬄine Learning Effects

**FIGURE 4:**
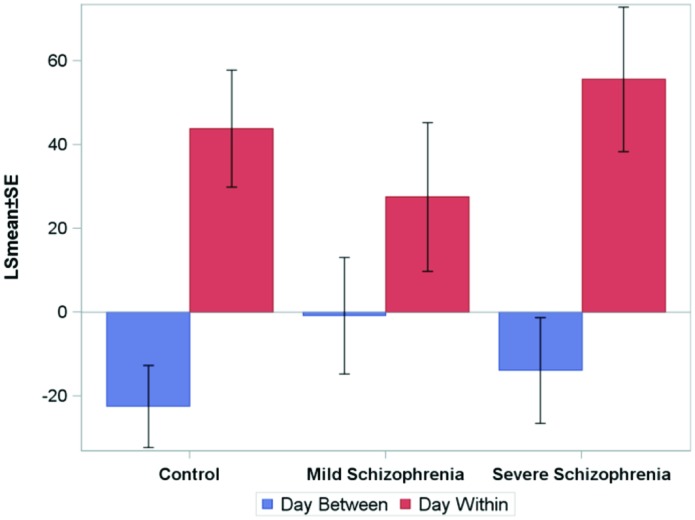
**The average (across the first 10 days of practice, across subjects) of the within-day and the between-days RT-gains for the three groups.** Negative numbers imply “forgetting.” The average time that passed between consecutive practice days was 10.5±2, 6+0.6, and 5±1, days for the CONT, MILD, and SEV groups, respectively.

To analyze the online (within session) and oﬄine (between days) practice effects of the groups, we defined: (i) **within-day RT gain** (within-day) as the difference between the LSmean RT of the first and the last block on a given practice day, averaged across subjects; (ii) **between-day RT gains** (between-days) as the difference between the LSmean RT of the last block on a given day, and the LSmean RT of the first block on the following practice day. **Figure 4** shows the average (across the first 10 days of practice) of the within-day and the between-day RT gains for the three groups.

For all groups, positive RT gains were found within-day (online effects), but not between-days, where small forgetting effects were found (negative off line effects). The within-day and between-day parameters were modeled using repeated-measures ANOVA models. Within-day and between-days were modeled as a function of day, group, and the group-by-day interaction term. We found that for the within-day effect, no statistically significant difference was found between the groups, but the interaction term was significant, implying that the pattern of change over days of the within-day speed gain differs between the groups. For the between-days effect, the analysis revealed no statistically significant difference between the groups and the interaction term was not significant. No statistically significant correlation between the number of days that passed between consecutive practice days and the between-days RT gain was found.

### The Effect of Practice on the Intra-Subject Variability and the Smoothness of Performance

One characteristic of RTs for different populations and disorders is the intra-subject RT variability. We noted that in the initial stage of learning some of the severe patients had very large RT variability, with very long pauses (up to 22 s; >25-fold their initial average RT) and fragmented performance. However, this abnormal variability largely disappeared with practice, reaching normal levels relative to the RT itself, which could be described as smoother performance. We analyzed this variability within blocks, using the CV (see Materials and Methods, Statistical Analysis). We found that the CV in the CONT and MILD groups increased a little between days 1 and 10 (from CV = 26.25% to CV = 34.61% for the CONT group; and from CV = 22.15% to CV = 32.20% for the MILD group). In the CONT and the MILD groups, this increase was probably due to subsequence learning (see **Figure 6**) and to a systematic reduction in the mean RT (**Figure 2**), with *SD* decreasing from 139 to 118 ms (CONT group), but not in the MILD group. In contrast, the CV for the SEV group was reduced to half between days 1 and 10 (from CV = 80.98% to CV = 38.43%), and the within-block *SD* was reduced from 674.7 ms on the first day of practice to 213.2 ms on the 10th day (see **Table 2**). Our data show that with practice, the long abnormal pauses that characterized the performance of the SEV group disappeared, yielding smoother, probably procedurally based performance (see section Evidence for Procedural Learning in Schizophrenia Patients).

**Table 2:** Intra-subject (within block) variability on practice days 1, 3 and 10 for each of the three groups.

Group	Day number	Within block *SD*	95% CI of within block *SD*	Within block CV%	95% CI of within block CV%
Control	1	139.524	[135.67–143.61]	26.25%	[17.50–33.75%]
Mild schizophrenia	1	122.589	[118.90–126.51]	22.15%	[14.15–29.15%]
Severe schizophrenia	1	674.698	[655.67–694.87]	80.98%	[24.77–104.3%]
Control	3	136.406	[132.77–140.24]	28.35%	[20.48–36.97%]
Mild schizophrenia	3	99.201	[95.985–102.64]	19.88%	[14.69–28.12%]
Severe schizophrenia	3	268.904	[261.32–276.94]	42.41%	[20.27–53.68%]
Control	10	118.170	[114.61–121.95]	34.61%	[23.36–50.05%]
Mild schizophrenia	10	125.879	[120.19–132.14]	32.20%	[18.59–73.22%]
Severe schizophrenia	10	213.238	[205.43–221.66]	38.43%	[30.04–42.42%]

### The Effect of Practice on the Inter-Subject Variability

We further explored the inter-subject variability of each group throughout the training sessions (see Materials and Methods, Statistical Analysis). The inter-subject variability was relatively high for the SEV group (a 2.4-fold range of individual subjects’ RT, 649–1566 ms); however, it was reduced to a 1.5-fold range on the eighth day of practice, similar to that found on the first day of practice in the CONT group. In contrast, the CONT group started with a relatively small inter-subject variability (455–657 ms) and reached a ninefold range of RTs (64–508 ms) on the eighth day of practice. The intra-subject variability of the MILD showed a similar tendency. The inter-subject variability was assessed by fitting several (one per day for days 1, 3, and 10) random effect models, where the RT was modeled with the subject entered as a random effect (see Materials and Methods, Statistical Analysis). The inter-subject variability throughout the days was compared via the coefficient of variation measure (CV), which is defined as the ratio of the model-estimated *SD* and the mean RT of the population). Using this analysis, we found that the between-subject variability in the control group almost doubled itself, increasing between day 1 and day 10 from CV = 28.4% to CV = 52.09%. The MILD group showed the same tendency: the between subject variability increased between day 1 and 10 from CV = 23.59% to CV = 47.27%. In contrast, the inter-subject variability in the SEV group decreased with training, by more than half between day 1 and day 10: from CV = 90.29% to CV = 41.29% (see **Table 3**).

**Table 3:** Between subject variability on practice days 1, 3, and 10 for each of the three groups.

Group	Day number	Between subject *SD*	95% CI of between subject *SD*	Between subject CV%	95% CI of between subject CV%
Control	1	150.94	[95.643–195.21]	28.40%	[18.59–35.64%]
Mild schizophrenia	1	130.51	[89.088–163.54]	23.59%	[15.61–30.05%]
Severe schizophrenia	1	752.28	[241.34–1158.4]	90.29%	[34.88–114.6%]
Control	3	143.88	[109.48–180.69]	29.90%	[21.93–37.93%]
Mild schizophrenia	3	142.41	[87.661–180.85]	28.54%	[17.05–41.23%]
Severe schizophrenia	3	338.28	[154.37–483.42]	53.36%	[29.28–65.52%]
Control	10	177.87	[125.84–200.26]	52.09%	[30.42–70.44%]
Mild schizophrenia	10	184.80	[88.293–203.84]	47.27%	[18.59–73.22%]
Severe schizophrenia	10	229.13	[153.46–271.23]	41.29%	[30.75–45.17%]

To summarize so far, we found that the SEV group was slower than the other groups, exhibited a different type of learning than did the CONT and MILD groups, and after several days of practice reached RT values that were within the range of the initial RTs of the other two groups. They also exhibited opposite tendencies regarding the pattern of inter- and intra- subject RT variability during learning. **Table 4** summarizes these results. No oﬄine learning effect was found in our study, pointing to implicit (as oppose to explicit) learning process (Robertson et al., 2004).

**Table 4:** The table summarizes the characteristics of the learning as a function of practice days, for the SEV (pink), and the CONT (blue) groups.

Group:	SEV			CONT		
Days:	1–3	4–8	9–14	1–3	4–8	9–14
Average RT msec On the first and the last days	854 ± 44		503 ± 50	521 ± 41		283 ± 44
Learning rate	F	S	S/N	N/S	F	S/N
Inter-subject variability	H		L	L		H
Intra-subject variability	H		L	L		H
Spatial-position-effect	X		√	√		X
Series-position-learning	X	X	X	X	x/√	√

*The SEV group started from fast (F) learning rate on the first 3 days, and moved to slow (S) and No-change (N) rate; and from High (H) inter and intra subject variability to Lower (L) variability. The CONT group showed an opposite tendency. Note that on the last day of practice, the SEV group reached the same characteristics that represent the performance of the CONT group.*

We now turn to investigate the different aspects of the learning, staring with the statistical learning as reflected by spatial specific learning.

### Spatial Position Specific Learning

In this section, we analyzed the learning-related improvement of the RT, for the different spatial positions. For this purpose we grouped together trials that shared the same spatial position throughout the trained sequence (3 repetitions in a trained sequence, 24 repetitions in a block), and tested their mean RT as a function of training. Given our experimental settings, and the theoretical arguments presented in the introduction, we hypothesized that statistical learning should result in faster responses to the cues at the central spatial positions (p2 and p3) and slower RTs to the peripheral positions (p1 and p4). No RT differences are expected between positions that share similar spatial eccentricity. Learning the serial order of the sequence, or parts of it, should reduce or diminish these spatial effects (see the Introduction).

**FIGURE 5:**
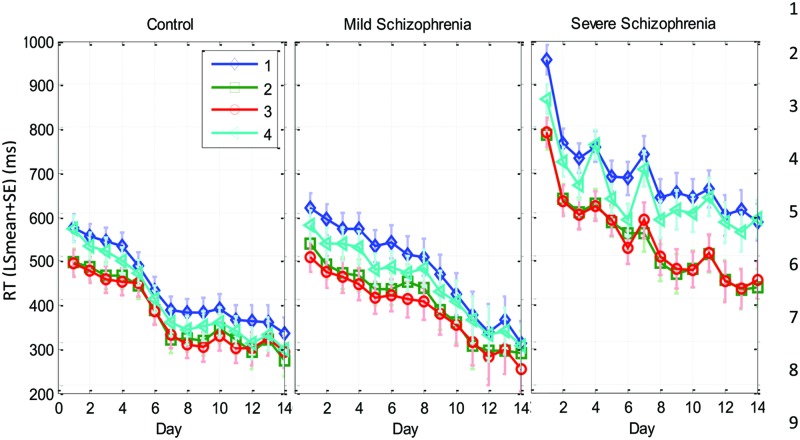
**Spatial position specific learning.** The mean series RT across participants as a function of day for the three experimental groups (SEV, *n* = 8, MILD, *n* = 7, CONT, *n* = 9) and for the four spatial positions (1–4 in different colors) as estimated from the model (see Materials and Methods). The error bars denote the residual *SD*. Note that the SEV group showed increasingly faster RTs for the central compared to the peripheral positions with practice. In comparison, the RTs of all positions for the CONT and MILD groups converged with practice.

**Figure 5** plots the LSmean RT over time for each spatial position (p1–p4) by group. Using repeated measures analysis of variance, we compared the groups and within the groups, per day, by spatial position. Before fitting the model, we averaged the 24 trials of each spatial position within blocks on each day. Thus, we modeled the (mean) RT as a function of group, spatial position, day, and the day × group × spatial position interaction term. Day was entered as a random effect where the blocks within day, per subject were treated as the repeated measure. Based on this analysis, we found that the RTs were affected by eccentricity (central vs. peripheral positions), as hypothesized here by probability-related considerations. However, this effect changed as a function of training and group.

On the first day of practice the CONT group exhibited a significant RT difference (>76 ms, *p* < 0.0001) in all four combinations of central vs. peripheral position comparisons. The |RT difference| between responses to cues with the same spatial eccentricities (i.e., p1, p4, and p2, p3) was <4 ms (n.s.), with faster RTs for the central spatial positions. This probability-related effect (see the Introduction) faded with practice, reaching similar mean RTs for all spatial positions on the last day of practice. In contrast, it took the SEV group 8–9 practice days to reach the above spatial position-specific effect, with a significant RT difference in all four combinations of central vs. peripheral position comparisons (|RTdiff| > 135 ms; *p* < 0.0001 for cross eccentricity comparisons; |RT diff| < 17 ms (n.s.) for the same eccentricity comparisons). This probability-related effect persisted until the last day of practice. (See Supplementary Table S1 for full details).

The analysis of the MILD group’s data showed a similar but weaker effect compared with that found in the CONT group (see **Figure 5** and Supplementary Table S1). On the first day of practice, the MILD group showed a significant RT difference in all four combinations of central vs. peripheral positions, with faster RTs for the central spatial positions. (RT difference > 73 ms, *p* < 0.0001 for three cross eccentricity comparisons, and RT difference = 43 ms, *p* = 0.015 for the p3, p4 combination.) The RT difference between the responses to the two central cues was n.s. (*p* < 0.093), and the RT difference between the responses to the two peripheral cues was significant (*p* = 0.024). This probability-related effect faded with practice, reaching non-significant differences in RTs for all spatial positions on the last day of practice (see Supplementary Table S1).

### Serial Order Learning

In this section, we analyzed the learning-related improvement in the RT, for the different series positions. For this purpose, for each subject we grouped trials that shared the same serial position (1–12) throughout each block (eight repetitions per position per block), and tested their mean RT (across the blocks per day) as a function of the training day. Our aim was to see if and how subsequence learning develops in the SRT task, and how spatial position learning (which could be related to statistical learning) relates to series-position learning (which could be related to chucking processes).

**FIGURE 6:**
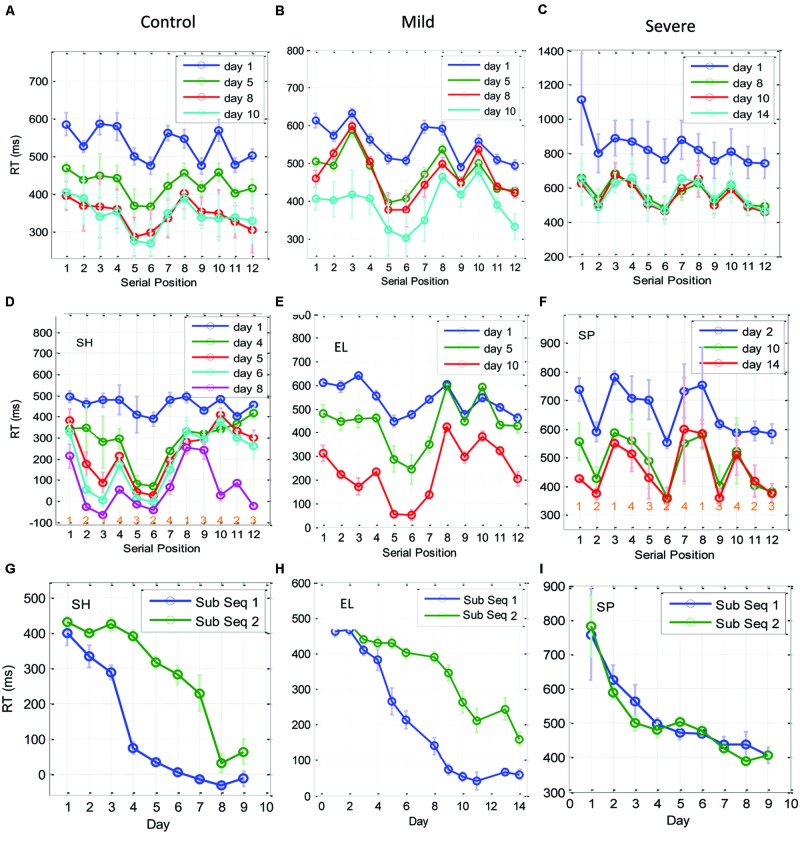
**Learning of subsequences as a function of training. (A–C)** The temporal dynamics of the RT for each of the 12 elements in the trained sequence, plotted for the CONT **(A)**, the MILD **(B)**, and the SEV **(C)** groups. The *x*-axis represents the serial position (1–12) in the sequence, with RT data averaged across blocks in a day (eight repetitions in a block, three blocks in a day) and then averaged across subjects. Error bars denote 1 *SE* of the mean across subjects. **(D–F)** are individual examples from one control subject, one mild and one severe patients, respectively. At the bottom of sub-figures **(D,F)** we introduced for each serial position the related spatial position to be pressed in the experiment. It can be seen that during the initial phase of training (CONT group) and on the last days of training (SEV groups) the RTs for the central positions were faster than the responses to the peripheral positions, regardless of their serial position. With training, the CONT group learned a subsequence (series position 5–6), and performed it better than all the other subsequences, including the subsequence at positions 11–12, which included the same spatial-position in a different order (‘1-2-1-4-3-2-4-3-1-4-2-3’). These effects were less clear for the MILD group; however, this group did display temporal dynamics similar to the CONT group. Since the subjects learned the subsequences at a different rate and order, we present here three individual examples for one control, one mild, and one severe patient. **(G–I)** Three examples of individual RT learning curves of two sub-parts of the trained sequence [serial positions 5,6, (sub Seq 1) and serial positions 11,12 (sub Seq 2)], for a control subject **(G)**, a mild patient **(H)**, and a severe patient **(I)**. The two subsequences shared the same spatial positions (p2, p3), but differed in their temporal order. Data were averaged across three blocks per day × two serial positions (*N* = 6); error bars denote 1 *SE* of this mean.

**Figure 6** shows the per serial position mean RTs on the first day of practice, and on three other representative practice days at later stages of learning for the CONT, MILD, and the SEV groups. We found that on the first day of practice the CONT group exhibited a regular pattern of spatial-position related temporal behavior, with a clear difference between series positions that were related to central spatial positions (series positions 2, 5, 6, 9, 11, and 12), and positions that were related to peripheral spatial positions (series positions 1,2,4,7,8, and 10; **Figure 6A**). The SEV group started much slower than the CONT group, and needed several days of training to develop a clear spatial position-related pattern of performance with faster RTs for central spatial positions, regardless of their serial position (**Figure 6C**). The MILD group (**Figure 6B**) displayed a less clear effect of spatial-position-specific learning on the first day of practice. These results are consistent with the results and analyses of the previous two sections (see the Discussion).

With practice, the CONT (but not the SEV) group changed from a spatial-position-specific pattern to a series-related specific performance, where performance at positions 5 and 6 were similar to each other and were found to be systematically faster than all the other serial positions, including positions 11–12, which shared with them the same spatial positions (‘1214**32**4134**23**’; see individual examples in **Figures 6G–I** for one CONT, one MILD, and one SEV subject, respectively). We interpret this finding as evidence for chunking learning (see the Discussion). Since different subjects developed this type of learning at a different learning rate, implicitly dividing the sequence into different fragments (or chunks) on different days, the group’s average RT (across subjects) could mask the fine details of this type of learning. Thus, we show in **Figures 6D–F** examples of chunking in one control, one mild, and one severe patient. As shown, the control subject and the mild patient (but not the severe patient) developed sequence-specific learning gradually, via subsequence learning. Moreover, a direct comparison of the average RT for the fastest and most salient positions (5 and 6), to positions 11, 12, which share with it the same spatial positions (2 and 3), shows that for the control subject and the mild patient, but not for the severe patient, the two subsequences were learned at a different rate, pointing to chunking learning (**Figures 6G–I**). The individual examples demonstrate how sequence knowledge can develop via a gradual chucking process (e.g., **Figure 6D**).

### Transfer Test

Although the main emphasis of the current study was on investigating statistical and subsequence learning, we conducted an additional test, in a subset of the patients, to determine the specificity of the learning in relation to the trained sequence (S12). Only patients that had undergone extensive training (>7 days) were tested: four severe patients, one mild patient, and seven controls. Three control subjects, who exhibited a highly significant learning gain after the first test for transfer, performed another test for transfer at a later, advanced practice stage, which was used to assess their transfer effect. Specificity of learning was quantified by determining the percentage of transfer of the learning gain from the trained to the new sequence (see Materials and Methods), with 100% transfer indicating that no sequence-specific learning had occurred.

**FIGURE 7:**
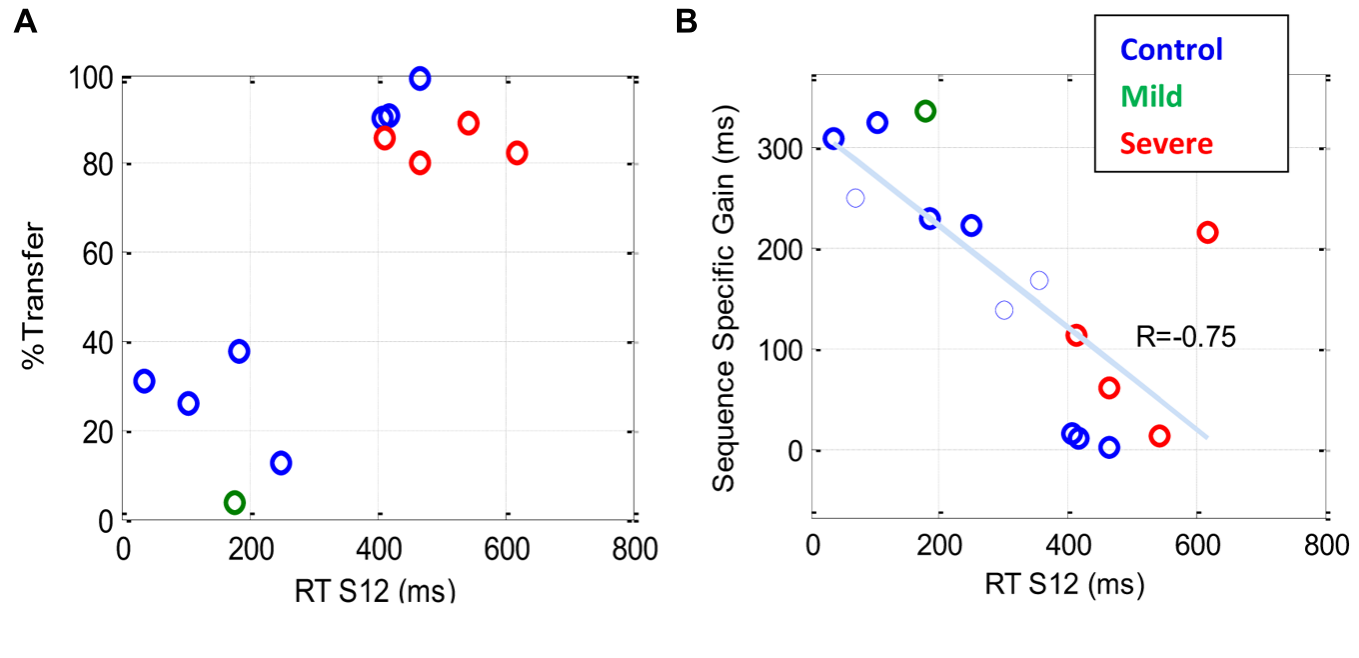
**The sequence specificity of training following extensive practice.** Sequence specificity was assessed by measuring series RT on a new sequence (R12) following a multi-day period of training on the first sequence (S12). The plots show a scatter representation of different individuals for two specificity measures plotted against the general level of performance [RT (S12)] before the transfer test. **(A)** The percentage of transfer of learning, one data point (latest measure) per subject **(B)** Sequence-specific gain (see Materials and Methods); note that in **(B)**, two (rather than one) data points are shown for each of three subjects who were tested twice, the first of whom is shown as a thin line. Both graphs show that the speed of performance in all subjects, both patients and controls, is inversely correlated with the specificity of learning, with faster being more specific.

**Figure 7A** shows the percentage of transfer of learning from the trained (S12) to the new (R12) sequence. The results indicate a dependency between the percentage of transfer and the general speed of performance (RT of S12): a high (>80%) transfer for the slow performers (four severe patients and three controls) and a small (<40%) transfer for the faster performers. **Figure 7B** shows the sequence-specific gain [the difference between RT(S12) and RT(R12)] plotted against RT(S12) for all the transfer tests that were conducted. The trend line shows a negative correlation (*R* = –0.75) between the sequence-specific gain and the trained RT, with slower individuals (patients and controls) being less specific. Taken together, these results suggest that the speed of performance in all subjects indicates the specificity of learning, with faster being more specific.

## Discussion

We studied the time course and characteristics of SEQL in schizophrenia (severe and mild) patients and control subjects. All participants practiced the task in short sessions over many days, (5–14 sessions; with some reaching over 20 practice sessions, on different days). The sequence was introduced implicitly via visual cues on a touch tablet, which triggered a sequence of lateral movements to be learned (Richard et al., 2009). The data show a similar magnitude of learning (speed gains) across days for all three groups, including the severe schizophrenia patients (∼270 ms speedup, **Figure 2**). However, the groups differed in overall speed: comparable RT was found for the mild patients and controls (∼300 ms after practice), but slower RT was found for the severe patients (550 ms after practice). By analyzing the data according to its spatial (**Figure 5**) and temporal (**Figure 6**) components, we provide evidence for two types of procedural learning: an initial visual-motor statistically related learning process (**Figure 5**), and later, series-related, subsequence learning (**Figure 6**), which typically characterizes the SRT paradigm. Next, we will discuss the different learning types and the way that patients can be distinguished from controls.

### Evidence for Two Stages in Sequence Learning

Statistical learning was suggested to be a component of SEQL (Perruchet and Pacton, 2006). Using the SRT paradigm, several studies (Stadler, 1992; Reed and Johnson, 1994) have shown that SRT performance is sensitive to the statistical relationships between trials and transitions. Moreover, (Hunt and Aslin, 2001) have shown that in a given SEQL task, subjects may implicitly learn several statistics in parallel. These important demonstrations leave open the question of whether statistical learning and subsequence learning are independent processes. This fundamental question is the subject of a debate that goes beyond the specific realm of the visual-motor SRT task (Meulemans and Van der Linden, 2003; Perruchet and Pacton, 2006). Theoretically, statistical computations and chunk formation (here, subsequence learning) could be two independent processes that may automatically and simultaneously be activated (Meulemans and Van der Linden, 2003; Perruchet and Pacton, 2006). In the current study we explored the evolution in time of statistical and SEQL in a SRT task over a prolonged period of time, and thus we could examine this fundamental question. Our results clearly showed that statistical learning and subsequence learning are two stages in SEQL. We found that statistically related learning is evident for slow responses, around 550 ms, whereas subsequence learning is evident at faster RTs (around 300 ms; **Figures 5** and **6**). Note that the shape of the learning curves in **Figures 2** and **3** provides independent and complementary indications of the two stages of learning, as will be discussed next in Section “The interpretation of slow performance.” Our results also showed that the spatial-position-specific learning effect can be obtained very quickly, within the first day, block, or even the first cycle, as found for the CONT and MILD groups. These groups (but not the SEV group with the slower SEQL rate, **Figures 5** and **6**) progressed to the next stage of learning where they learned fragments of the sequence, and performed them faster than the others (**Figure 6**). These subsequences depended on the temporal position in the series and not on the spatial position of the cue. Importantly, the first subsequence that is learned (**Figure 6**) is typically based on the fastest subsequence temporal positions (5 and 6) whose speed advantage is due to implicit statistical considerations. The hypothesis that these findings suggest that the two learning processes are limited by different time constraints will be discussed in the next section. In summary, our findings support the prevalent information processing models in oral (Saffran, 2001; Perruchet and Pacton, 2006) and visual-scene analysis (Fiser and Aslin, 2005; Perruchet and Pacton, 2006) in which statistical computations and chunk formation are considered as two successive steps in information processing and SEQL, with chunks inferred from prior statistical computations (Perruchet and Pacton, 2006).

### Temporal Constraints on Sequence Learning

There are different temporal constraints inherent in the two stages of SEQL discussed above. Whereas theoretically, the visual-motor learning stage can be obtained by creating simultaneous pair wise associations between visual and motor cell activations, the formation of sequence-specific predictions requires the integration of information from three successive trials, owing to the properties of the SOC sequence. It is reasonable to assume that the need to form associations among three successive elements is limited by the capacity and temporal characteristics of the implicit working memory of the participants, and by the temporal properties of the mechanism that creates these associations. For example, a possible mechanism for both learning types may rely on Hebbian associations, which require the activation of pairwise cell assemblies in a certain (small) time window (e.g., Caporale and Dan, 2008). These considerations suggest that in the absence of declarative knowledge, statistical learning can be activated at slower RTs compared with sequence-specific learning. This conjecture is supported by the current finding that the statistical learning is evident here in a time window of ∼500 ms (plus the 200 ms of the RSI) or even higher, whereas sequence-specific learning becomes effective below an average RT of 500 ms (**Figures 5–7**). Given these constraints, the statistically related learning could precede and possibly enable the learning of the sequence. In support of this argument, we noted that the first subsequence that is learned is typically based on the fastest subsequence temporal positions (5 and 6) whose speed advantage is due to implicit statistical considerations (**Figure 6**).

### Spatial-Position Specific Learning as a Statistical Learning Process

In the Introduction, we hypothesized that under the constraints of our experiment, a statistical learning component should appear as a spatial position-specific learning effect, with faster responses to the central spatial positions relative to the lateral positions, regardless of their serial order in the sequence. In accordance with this assumption, the data showed that at an average RT of around 550 ms, the trained subjects of all three groups responded faster to the central positions than to the peripheral ones (**Figure 5**; **Figure 6A** day1; **Figure 6C** day > 7), regardless of their position in the sequence. The finding that statistical-related performance is found above a certain speed limit supports the notion that it reflects the activation of a probability-based internal program. According to our interpretation in the Section “Introduction,” the advantage of the central positions may suggest the existence of a probability-based motor plan that enables the preparation and even the initiation of lateral movements toward the location with the highest probability. Such a program can set the direction of the next movement when initiated from the lateral positions, and may set the velocity of the movement according to the average step size in the task (1–2 squares on average). Being a probability-based program, its performance is limited, since the final step of the movement (the actual choice) depends on the actual visual cue (Willingham et al., 1989).

### The Interpretation of Slow Performance

One salient finding in our study is that the SEV group performed slower than the other two groups but had a similar overall gain of speed (∼270 ms), in accordance with previous work (Green et al., 1997). A naïve interpretation would suggest that similar speed gains reflect similar learning processes. However, a closer inspection reveals that the SEV group had different performance characteristics and a different time course of learning, with a paradoxically faster speed improvement during the initial practice days. Specifically, during the first 3 days of practice the SEV group achieved the majority of its speed gains (∼200/270 ms) and its average RT improved by 24% (**Figure 2**). In contrast, in this initial stage of practice, the other two groups exhibited much lower gains of speed (∼52/270 ms) and its average RT improved by 9–10% (See also individual examples, **Figure 3**). If one ignores the overall speed level of the groups, the finding of a higher initial learning rate for the severe patients could suggest that the SEV patients have a faster learning rate than do the controls. This suggestion could be surprising, given the various cognitive, memory, and motivation deficits that characterize severe schizophrenia, as well as the previous evidence for abnormal learning in these patients (Vohringer et al., 2013). A possible explanation is that the learning rate of the severe patients is in fact slower than that of the other two groups, with a prolonged initial statistics-related learning phase (**Figure 5**). This suggestion is strongly supported by the analysis we made in this study (for a summary, see **Table 4**), which suggests that the SEV group developed statistical-related visual-motor performance, whereas the CONT group moved from a fast stage of statistical learning to subsequence (sequence specific) learning (**Figures 5–7**). Our results support previous suggestions that practice does not necessarily make perfect, and that a limiting factor in a given context could be the period of plastic changes (a few sessions), and the amount of progress in a given learning context (Adini et al., 2002). It also suggests that the absolute (and not just the relative) level of performance is an important factor that should be taken into account when studying implicit learning.

### Sequence-Specific Learning in Schizophrenia Patients

Our results extend the findings of many previous studies that showed conflicting results regarding the ability of schizophrenia patients to learn the temporal order of the trained sequence (Green et al., 1997; Perry et al., 2000; Marvel et al., 2007; Foerde et al., 2008; Siegert et al., 2008; Remillard, 2014). We found that some of the patients from the MILD group did exhibit subsequence learning. In comparison, the patients from the SEV group, who were tested for transfer with another sequence, exhibited little or no sequence-specific learning, since above 80% of their learning was transferred to a new SOC sequence (**Figure 7**). Instead, they displayed an evolution of statistical learning throughout the practice days. Our findings are in accordance with previous suggestions that procedural learning in the SRT can be divided into separate learning processes, one of which is sequence-specific and the other which is not (Knopman and Nissen, 1991; Green et al., 1997).

### Evidence for Procedural Learning in Schizophrenia Patients

In many studies, schizophrenics have been found to be slower and more variable than normal controls (Schwartz et al., 1989). Since RT appears to have trait-like properties in schizophrenia patients (Schwartz et al., 1989), the diversity of assumptions and theoretical arguments that are represented within the boundaries of RT experiments is immense (Nuechterlein, 1977), including, for example, “dissociation from environmental stimuli, lower motivation, less intense concentration of attention or inability to attain a high level of preparation” (Rodnick and Shakow, 1940). In our study, as in previous studies (e.g., Green et al., 1997), the severe (but not the mild) patients exhibited high intra-block RT variability, with occasional long pauses (up to 22 s; >25-fold of their initial RT). Importantly, with practice, these pauses were eliminated, and the performance became faster and smoother (the intra-block variability halved itself), leading to similar mean and median RT values. Since our measured RT reflects the time needed for correct responses, our results also show (indirectly) a decrease in the rate of error, a gain of speed and smoothness, all of which are indications of the development of procedural (skill) learning (e.g., Shmuelof et al., 2012). In light of our finding regarding statistical and subsequence learning in the different groups, we suggest that given prolonged practice, schizophrenia patients, including those patients with high, predominantly negative symptoms and rigid behavior, can acquire and retain different types of procedural knowledge and new, sensory-motor, cognitive-related skills. These newly acquired procedures may lead to relatively fluent performance. However, the procedural knowledge that underlies a new skill may differ among subsets of patients and healthy controls.

The ability of schizophrenia patients to acquire new procedures is supported by previous studies that found evidence of preserved skill learning in schizophrenia, as assessed by the artificial grammar paradigm (Danion et al., 2001; Horan et al., 2008), the Tower-of-Hanoi tasks (Goldberg et al., 1990), and the rotary pursuit task (De Picker et al., 2014). Note that in all of these studies, as was found here, the patients needed more practice, sometimes over multiple days, to reach performance comparable to that of healthy controls (Goldberg et al., 1990; Danion et al., 2001; De Picker et al., 2014).

### Why do Severe Schizophrenia Patients have Deficits in Sequence-Specific Learning?

Previous studies show conflicting results regarding the ability of schizophrenia patients to develop sequence-specific learning in the SRT task (Green et al., 1997; Perry et al., 2000; Marvel et al., 2007; Foerde et al., 2008; Siegert et al., 2008). Researchers thus concluded that at least some of the schizophrenia patients suffer from deficits in SRT procedural learning (Green et al., 1997). Here we tested the possibility that the inability of the patients to develop sequence-specific learning is due to the short practice period with the task (i.e., all the training was in a single session). We found that the patients could be divided into two sub-groups. Of these, the group of patients with the severe predominantly negative symptoms (some of them with rigid, robotic-like behavior) exhibited very little sequence-specific learning (**Figure 7**), and did not exhibit subsequence learning (**Figures 5** and **6**). In contrast, their learning was dominated by statistically related learning, even after months of practice consisting of 2–3 sessions per week (**Figures 5** and **6**). Possible explanations derived from previous studies include deficits in their implicit working memory (Haenschel and Linden, 2011), and/or deficits in their cortico-cerebellar loop (Green et al., 1997). Our results suggest an alternative explanation, in which an initial temporal constraint, imposed by slow performance, prevents higher order associations that are critical for learning the sequence. With practice, however, the patients become fast enough to counteract this temporal constraint, but at that stage they reach a stable non-plastic state owing to prolonged practice with a constant context (Adini et al., 2002). This explanation suggests that changing the sequence or other contextual elements should enable or promote learning.

## Summary and Conclusion

We found that prolonged practice, with short (few minutes) sessions over multiple days was enough to develop and consolidate procedural learning having similar gain in all subjects, both schizophrenia patients and healthy controls. The statistical learning component was found to be intact in the schizophrenia patients. However, those patients with high, predominantly negative symptoms (i.e., the severe patients) were slower to acquire the spatial position-specific statistics, possibly due to their large temporal variability at the initial stages of learning. Importantly, after reaching a saturation level at RT similar to the initial speed of the controls, some severe patients could quickly improve their performance when the sequence length and complexity were reduced, thus demonstrating that their performance limit was not due to an inability to move faster or to learn subsequences. Future studies should explore the possibility that long sequences could be learned by severe schizophrenia patients, using a cascade of short subsequences.

The finding that patients with severe schizophrenia can learn and retain procedural skills, while exhibiting different performance characteristics that can be measured using a portable bedside device (tablet), has interesting clinical implications. For example, the paradigm could be used for an objective clinical assessment, since the performance characteristics (with or without learning) differentiated those patients with mild and severe predominantly negative symptoms. Moreover, this study presents a possible method for bypassing the initiation and motivational deficits and the difficulties in performing basic, everyday procedures that characterize severe schizophrenia.

## Conflict of Interest Statement

The Associate Editor Srikantan S. Nagarajan declares that depite of being affiliated with the same institution as the Review Editor Leighton B. Hinkley, the review process was handled objectively. The authors declare that the research was conducted in the absence of any commercial or financial relationships that could be construed as a potential conflict of interest.
