# Supplementary material for: The time course and characteristics of procedural learning in schizophrenia patients and healthy individuals
Source: Front Hum Neurosci. 2015 Sep 1;9:475. doi: 10.3389/fnhum.2015.00475 (PMC4555022; doi:10.3389/fnhum.2015.00475)
Supplement: Supplementary file 1 [file Table_1.DOC]

| **Table S1** | | | | **LSmean Difference** | **DF** | **t Value** | **P-Value** | **Lower 95% CL** | **Upper 95% CL** |
| --- | --- | --- | --- | --- | --- | --- | --- | --- | --- |
| **Day Number** | **Group** | **Spatial Position** | **Spatial Position** | 80.28 | 2756 | 4.95 | <.0001 | 48.48 | 112.08 |
| **1** | **Control** | **1** | **2** |  |  |  |  |  |  |
|  |  |  | **3** | 82.95 | 2756 | 5.11 | <.0001 | 51.15 | 114.75 |
|  |  |  | **4** | 3.65 | 2756 | 0.23 | 0.8219 | -28.15 | 35.45 |
|  |  | **2** | **3** | 2.67 | 2756 | 0.16 | 0.8693 | -29.13 | 34.47 |
|  |  |  | **4** | -76.63 | 2756 | -4.73 | <.0001 | -108.43 | -44.83 |
|  |  | **3** | **4** | -79.30 | 2756 | -4.89 | <.0001 | -111.10 | -47.50 |
|  | **Mild Schizophrenia** | **1** | **2** | 83.14 | 2756 | 4.70 | <.0001 | 48.44 | 117.83 |
|  |  |  | **3** | 112.87 | 2756 | 6.38 | <.0001 | 78.17 | 147.56 |
|  |  |  | **4** | 40.07 | 2756 | 2.26 | 0.0236 | 5.38 | 74.77 |
|  |  | **2** | **3** | 29.73 | 2756 | 1.68 | 0.0930 | -4.97 | 64.43 |
|  |  |  | **4** | -43.07 | 2756 | -2.43 | 0.0150 | -77.76 | -8.37 |
|  |  | **3** | **4** | -72.80 | 2756 | -4.11 | <.0001 | -107.49 | -38.10 |
|  | **Severe Schizophrenia** | **1** | **2** | 173.58 | 2756 | 10.49 | <.0001 | 141.13 | 206.03 |
|  |  |  | **3** | 168.60 | 2756 | 10.19 | <.0001 | 136.14 | 201.05 |
|  |  |  | **4** | 90.36 | 2756 | 5.46 | <.0001 | 57.91 | 122.82 |
|  |  | **2** | **3** | -4.98 | 2756 | -0.30 | 0.7634 | -37.44 | 27.47 |
|  |  |  | **4** | -83.22 | 2756 | -5.03 | <.0001 | -115.67 | -50.76 |
|  |  | **3** | **4** | -78.23 | 2756 | -4.73 | <.0001 | -110.69 | -45.78 |
| **2** | **Control** | **1** | **2** | 70.27 | 2756 | 4.67 | <.0001 | 40.75 | 99.80 |
|  |  |  | **3** | 78.38 | 2756 | 5.21 | <.0001 | 48.86 | 107.91 |
|  |  |  | **4** | 20.67 | 2756 | 1.37 | 0.1700 | -8.86 | 50.19 |
|  |  | **2** | **3** | 8.11 | 2756 | 0.54 | 0.5903 | -21.42 | 37.63 |
|  |  |  | **4** | -49.61 | 2756 | -3.29 | 0.0010 | -79.13 | -20.08 |
|  |  | **3** | **4** | -57.72 | 2756 | -3.83 | 0.0001 | -87.24 | -28.19 |
|  | **Mild Schizophrenia** | **1** | **2** | 103.74 | 2756 | 5.86 | <.0001 | 69.04 | 138.43 |
|  |  |  | **3** | 122.57 | 2756 | 6.93 | <.0001 | 87.88 | 157.27 |
|  |  |  | **4** | 56.57 | 2756 | 3.20 | 0.0014 | 21.88 | 91.27 |
|  |  | **2** | **3** | 18.84 | 2756 | 1.06 | 0.2872 | -15.86 | 53.53 |
|  |  |  | **4** | -47.16 | 2756 | -2.67 | 0.0077 | -81.86 | -12.47 |
|  |  | **3** | **4** | -66.00 | 2756 | -3.73 | 0.0002 | -100.70 | -31.30 |
|  | **Severe Schizophrenia** | **1** | **2** | 129.07 | 2756 | 7.80 | <.0001 | 96.61 | 161.52 |
|  |  |  | **3** | 133.21 | 2756 | 8.05 | <.0001 | 100.75 | 165.66 |
|  |  |  | **4** | 42.62 | 2756 | 2.57 | 0.0101 | 10.17 | 75.07 |
|  |  | **2** | **3** | 4.14 | 2756 | 0.25 | 0.8026 | -28.32 | 36.59 |
|  |  |  | **4** | -86.45 | 2756 | -5.22 | <.0001 | -118.90 | -54.00 |
|  |  | **3** | **4** | -90.59 | 2756 | -5.47 | <.0001 | -123.04 | -58.13 |
| **3** | **Control** | **1** | **2** | 78.32 | 2756 | 5.02 | <.0001 | 47.72 | 108.92 |
|  |  |  | **3** | 88.32 | 2756 | 5.66 | <.0001 | 57.72 | 118.92 |
|  |  |  | **4** | 20.42 | 2756 | 1.31 | 0.1908 | -10.18 | 51.02 |
|  |  | **2** | **3** | 10.00 | 2756 | 0.64 | 0.5217 | -20.60 | 40.60 |
|  |  |  | **4** | -57.90 | 2756 | -3.71 | 0.0002 | -88.50 | -27.30 |
|  |  | **3** | **4** | -67.90 | 2756 | -4.35 | <.0001 | -98.50 | -37.30 |
|  | **Mild Schizophrenia** | **1** | **2** | 101.80 | 2756 | 5.33 | <.0001 | 64.33 | 139.28 |
|  |  |  | **3** | 109.92 | 2756 | 5.75 | <.0001 | 72.44 | 147.39 |
|  |  |  | **4** | 35.07 | 2756 | 1.84 | 0.0666 | -2.40 | 72.55 |
|  |  | **2** | **3** | 8.12 | 2756 | 0.42 | 0.6710 | -29.36 | 45.59 |
|  |  |  | **4** | -66.73 | 2756 | -3.49 | 0.0005 | -104.20 | -29.25 |
|  |  | **3** | **4** | -74.85 | 2756 | -3.92 | <.0001 | -112.32 | -37.37 |
|  | **Severe Schizophrenia** | **1** | **2** | 128.39 | 2756 | 7.76 | <.0001 | 95.94 | 160.85 |
|  |  |  | **3** | 132.25 | 2756 | 7.99 | <.0001 | 99.79 | 164.70 |
|  |  |  | **4** | 64.26 | 2756 | 3.88 | 0.0001 | 31.81 | 96.72 |
|  |  | **2** | **3** | 3.85 | 2756 | 0.23 | 0.8160 | -28.60 | 36.31 |
|  |  |  | **4** | -64.13 | 2756 | -3.87 | 0.0001 | -96.58 | -31.68 |
|  |  | **3** | **4** | -67.98 | 2756 | -4.11 | <.0001 | -100.44 | -35.53 |
| **4** | **Control** | **1** | **2** | 66.66 | 2756 | 4.27 | <.0001 | 36.06 | 97.26 |
|  |  |  | **3** | 82.63 | 2756 | 5.30 | <.0001 | 52.03 | 113.23 |
|  |  |  | **4** | 34.43 | 2756 | 2.21 | 0.0275 | 3.83 | 65.03 |
|  |  | **2** | **3** | 15.97 | 2756 | 1.02 | 0.3061 | -14.63 | 46.57 |
|  |  |  | **4** | -32.23 | 2756 | -2.07 | 0.0390 | -62.83 | -1.63 |
|  |  | **3** | **4** | -48.20 | 2756 | -3.09 | 0.0020 | -78.80 | -17.61 |
|  | **Mild Schizophrenia** | **1** | **2** | 109.30 | 2756 | 6.18 | <.0001 | 74.61 | 144.00 |
|  |  |  | **3** | 130.57 | 2756 | 7.38 | <.0001 | 95.87 | 165.26 |
|  |  |  | **4** | 44.53 | 2756 | 2.52 | 0.0119 | 9.83 | 79.23 |
|  |  | **2** | **3** | 21.26 | 2756 | 1.20 | 0.2296 | -13.43 | 55.96 |
|  |  |  | **4** | -64.77 | 2756 | -3.66 | 0.0003 | -99.47 | -30.08 |
|  |  | **3** | **4** | -86.04 | 2756 | -4.86 | <.0001 | -120.73 | -51.34 |
|  | **Severe Schizophrenia** | **1** | **2** | 134.49 | 2756 | 8.13 | <.0001 | 102.04 | 166.95 |
|  |  |  | **3** | 139.43 | 2756 | 8.42 | <.0001 | 106.97 | 171.88 |
|  |  |  | **4** | -4.83 | 2756 | -0.29 | 0.7705 | -37.28 | 27.62 |
|  |  | **2** | **3** | 4.93 | 2756 | 0.30 | 0.7657 | -27.52 | 37.39 |
|  |  |  | **4** | -139.32 | 2756 | -8.42 | <.0001 | -171.78 | -106.87 |
|  |  | **3** | **4** | -144.26 | 2756 | -8.72 | <.0001 | -176.71 | -111.80 |
| **5** | **Control** | **1** | **2** | 45.95 | 2756 | 3.45 | 0.0006 | 19.81 | 72.09 |
|  |  |  | **3** | 40.83 | 2756 | 3.06 | 0.0022 | 14.69 | 66.97 |
|  |  |  | **4** | 16.30 | 2756 | 1.22 | 0.2216 | -9.84 | 42.44 |
|  |  | **2** | **3** | -5.12 | 2756 | -0.38 | 0.7010 | -31.26 | 21.02 |
|  |  |  | **4** | -29.65 | 2756 | -2.22 | 0.0262 | -55.79 | -3.51 |
|  |  | **3** | **4** | -24.53 | 2756 | -1.84 | 0.0659 | -50.67 | 1.61 |
|  | **Mild Schizophrenia** | **1** | **2** | 99.32 | 2756 | 5.20 | <.0001 | 61.85 | 136.80 |
|  |  |  | **3** | 119.83 | 2756 | 6.27 | <.0001 | 82.35 | 157.30 |
|  |  |  | **4** | 54.84 | 2756 | 2.87 | 0.0041 | 17.37 | 92.32 |
|  |  | **2** | **3** | 20.50 | 2756 | 1.07 | 0.2835 | -16.97 | 57.98 |
|  |  |  | **4** | -44.48 | 2756 | -2.33 | 0.0200 | -81.96 | -7.01 |
|  |  | **3** | **4** | -64.98 | 2756 | -3.40 | 0.0007 | -102.46 | -27.51 |
|  | **Severe Schizophrenia** | **1** | **2** | 108.06 | 2756 | 6.25 | <.0001 | 74.16 | 141.96 |
|  |  |  | **3** | 102.56 | 2756 | 5.93 | <.0001 | 68.66 | 136.46 |
|  |  |  | **4** | 53.27 | 2756 | 3.08 | 0.0021 | 19.38 | 87.17 |
|  |  | **2** | **3** | -5.50 | 2756 | -0.32 | 0.7505 | -39.40 | 28.40 |
|  |  |  | **4** | -54.79 | 2756 | -3.17 | 0.0015 | -88.69 | -20.89 |
|  |  | **3** | **4** | -49.29 | 2756 | -2.85 | 0.0044 | -83.19 | -15.39 |
| **6** | **Control** | **1** | **2** | 47.23 | 2756 | 3.14 | 0.0017 | 17.71 | 76.76 |
|  |  |  | **3** | 48.14 | 2756 | 3.20 | 0.0014 | 18.61 | 77.66 |
|  |  |  | **4** | 20.30 | 2756 | 1.35 | 0.1776 | -9.22 | 49.83 |
|  |  | **2** | **3** | 0.90 | 2756 | 0.06 | 0.9521 | -28.62 | 30.43 |
|  |  |  | **4** | -26.93 | 2756 | -1.79 | 0.0738 | -56.45 | 2.59 |
|  |  | **3** | **4** | -27.83 | 2756 | -1.85 | 0.0646 | -57.36 | 1.69 |
|  | **Mild Schizophrenia** | **1** | **2** | 110.35 | 2756 | 6.09 | <.0001 | 74.80 | 145.90 |
|  |  |  | **3** | 124.31 | 2756 | 6.86 | <.0001 | 88.76 | 159.86 |
|  |  |  | **4** | 58.28 | 2756 | 3.21 | 0.0013 | 22.73 | 93.83 |
|  |  | **2** | **3** | 13.96 | 2756 | 0.77 | 0.4413 | -21.59 | 49.51 |
|  |  |  | **4** | -52.07 | 2756 | -2.87 | 0.0041 | -87.62 | -16.52 |
|  |  | **3** | **4** | -66.03 | 2756 | -3.64 | 0.0003 | -101.58 | -30.48 |
|  | **Severe Schizophrenia** | **1** | **2** | 129.17 | 2756 | 6.17 | <.0001 | 88.12 | 170.22 |
|  |  |  | **3** | 161.82 | 2756 | 7.73 | <.0001 | 120.77 | 202.87 |
|  |  |  | **4** | 97.14 | 2756 | 4.64 | <.0001 | 56.09 | 138.20 |
|  |  | **2** | **3** | 32.65 | 2756 | 1.56 | 0.1190 | -8.40 | 73.70 |
|  |  |  | **4** | -32.03 | 2756 | -1.53 | 0.1262 | -73.08 | 9.02 |
|  |  | **3** | **4** | -64.68 | 2756 | -3.09 | 0.0020 | -105.73 | -23.62 |
| **7** | **Control** | **1** | **2** | 69.56 | 2756 | 4.85 | <.0001 | 41.45 | 97.67 |
|  |  |  | **3** | 58.30 | 2756 | 4.07 | <.0001 | 30.19 | 86.40 |
|  |  |  | **4** | 28.73 | 2756 | 2.00 | 0.0451 | 0.62 | 56.84 |
|  |  | **2** | **3** | -11.26 | 2756 | -0.79 | 0.4321 | -39.37 | 16.84 |
|  |  |  | **4** | -40.83 | 2756 | -2.85 | 0.0044 | -68.94 | -12.73 |
|  |  | **3** | **4** | -29.57 | 2756 | -2.06 | 0.0392 | -57.68 | -1.46 |
|  | **Mild Schizophrenia** | **1** | **2** | 64.60 | 2756 | 2.76 | 0.0058 | 18.70 | 110.50 |
|  |  |  | **3** | 104.06 | 2756 | 4.45 | <.0001 | 58.16 | 149.96 |
|  |  |  | **4** | 43.41 | 2756 | 1.85 | 0.0638 | -2.49 | 89.30 |
|  |  | **2** | **3** | 39.46 | 2756 | 1.69 | 0.0919 | -6.44 | 85.36 |
|  |  |  | **4** | -21.19 | 2756 | -0.91 | 0.3653 | -67.09 | 24.70 |
|  |  | **3** | **4** | -60.66 | 2756 | -2.59 | 0.0096 | -106.55 | -14.76 |
|  | **Severe Schizophrenia** | **1** | **2** | 185.76 | 2756 | 7.94 | <.0001 | 139.86 | 231.65 |
|  |  |  | **3** | 153.49 | 2756 | 6.56 | <.0001 | 107.59 | 199.38 |
|  |  |  | **4** | 35.22 | 2756 | 1.50 | 0.1325 | -10.68 | 81.12 |
|  |  | **2** | **3** | -32.27 | 2756 | -1.38 | 0.1681 | -78.17 | 13.63 |
|  |  |  | **4** | -150.54 | 2756 | -6.43 | <.0001 | -196.44 | -104.64 |
|  |  | **3** | **4** | -118.27 | 2756 | -5.05 | <.0001 | -164.16 | -72.37 |
| **8** | **Control** | **1** | **2** | 58.01 | 2756 | 3.72 | 0.0002 | 27.41 | 88.60 |
|  |  |  | **3** | 73.66 | 2756 | 4.72 | <.0001 | 43.06 | 104.26 |
|  |  |  | **4** | 40.26 | 2756 | 2.58 | 0.0099 | 9.66 | 70.86 |
|  |  | **2** | **3** | 15.65 | 2756 | 1.00 | 0.3159 | -14.94 | 46.25 |
|  |  |  | **4** | -17.75 | 2756 | -1.14 | 0.2555 | -48.35 | 12.85 |
|  |  | **3** | **4** | -33.40 | 2756 | -2.14 | 0.0324 | -64.00 | -2.80 |
|  | **Mild Schizophrenia** | **1** | **2** | 71.86 | 2756 | 3.07 | 0.0022 | 25.96 | 117.76 |
|  |  |  | **3** | 101.95 | 2756 | 4.36 | <.0001 | 56.05 | 147.85 |
|  |  |  | **4** | 23.97 | 2756 | 1.02 | 0.3059 | -21.93 | 69.87 |
|  |  | **2** | **3** | 30.09 | 2756 | 1.29 | 0.1988 | -15.81 | 75.98 |
|  |  |  | **4** | -47.89 | 2756 | -2.05 | 0.0409 | -93.79 | -1.99 |
|  |  | **3** | **4** | -77.98 | 2756 | -3.33 | 0.0009 | -123.87 | -32.08 |
|  | **Severe Schizophrenia** | **1** | **2** | 149.60 | 2756 | 6.39 | <.0001 | 103.70 | 195.49 |
|  |  |  | **3** | 137.89 | 2756 | 5.89 | <.0001 | 91.99 | 183.79 |
|  |  |  | **4** | 49.56 | 2756 | 2.12 | 0.0343 | 3.66 | 95.46 |
|  |  | **2** | **3** | -11.70 | 2756 | -0.50 | 0.6171 | -57.60 | 34.19 |
|  |  |  | **4** | -100.03 | 2756 | -4.27 | <.0001 | -145.93 | -54.14 |
|  |  | **3** | **4** | -88.33 | 2756 | -3.77 | 0.0002 | -134.23 | -42.43 |
| **9** | **Control** | **1** | **2** | 67.98 | 2756 | 3.56 | 0.0004 | 30.51 | 105.46 |
|  |  |  | **3** | 80.46 | 2756 | 4.21 | <.0001 | 42.99 | 117.94 |
|  |  |  | **4** | 33.61 | 2756 | 1.76 | 0.0787 | -3.86 | 71.09 |
|  |  | **2** | **3** | 12.48 | 2756 | 0.65 | 0.5138 | -25.00 | 49.95 |
|  |  |  | **4** | -34.37 | 2756 | -1.80 | 0.0722 | -71.85 | 3.10 |
|  |  | **3** | **4** | -46.85 | 2756 | -2.45 | 0.0143 | -84.32 | -9.37 |
|  | **Mild Schizophrenia** | **1** | **2** | 81.26 | 2756 | 3.01 | 0.0027 | 28.26 | 134.26 |
|  |  |  | **3** | 92.07 | 2756 | 3.41 | 0.0007 | 39.07 | 145.07 |
|  |  |  | **4** | 37.81 | 2756 | 1.40 | 0.1619 | -15.18 | 90.81 |
|  |  | **2** | **3** | 10.81 | 2756 | 0.40 | 0.6892 | -42.19 | 63.81 |
|  |  |  | **4** | -43.44 | 2756 | -1.61 | 0.1081 | -96.44 | 9.55 |
|  |  | **3** | **4** | -54.25 | 2756 | -2.01 | 0.0448 | -107.25 | -1.26 |
|  | **Severe Schizophrenia** | **1** | **2** | 190.61 | 2756 | 7.05 | <.0001 | 137.61 | 243.61 |
|  |  |  | **3** | 179.97 | 2756 | 6.66 | <.0001 | 126.97 | 232.97 |
|  |  |  | **4** | 42.20 | 2756 | 1.56 | 0.1186 | -10.80 | 95.20 |
|  |  | **2** | **3** | -10.64 | 2756 | -0.39 | 0.6938 | -63.64 | 42.35 |
|  |  |  | **4** | -148.41 | 2756 | -5.49 | <.0001 | -201.41 | -95.41 |
|  |  | **3** | **4** | -137.77 | 2756 | -5.10 | <.0001 | -190.77 | -84.77 |
| **10** | **Control** | **1** | **2** | 45.63 | 2756 | 2.58 | 0.0100 | 10.94 | 80.33 |
|  |  |  | **3** | 63.19 | 2756 | 3.57 | 0.0004 | 28.50 | 97.89 |
|  |  |  | **4** | 31.88 | 2756 | 1.80 | 0.0717 | -2.82 | 66.57 |
|  |  | **2** | **3** | 17.56 | 2756 | 0.99 | 0.3210 | -17.13 | 52.26 |
|  |  |  | **4** | -13.75 | 2756 | -0.78 | 0.4370 | -48.45 | 20.94 |
|  |  | **3** | **4** | -31.32 | 2756 | -1.77 | 0.0769 | -66.01 | 3.38 |
|  | **Mild Schizophrenia** | **1** | **2** | 65.27 | 2756 | 2.41 | 0.0158 | 12.27 | 118.27 |
|  |  |  | **3** | 73.19 | 2756 | 2.71 | 0.0068 | 20.19 | 126.19 |
|  |  |  | **4** | 18.48 | 2756 | 0.68 | 0.4943 | -34.52 | 71.47 |
|  |  | **2** | **3** | 7.92 | 2756 | 0.29 | 0.7695 | -45.08 | 60.92 |
|  |  |  | **4** | -46.79 | 2756 | -1.73 | 0.0835 | -99.79 | 6.21 |
|  |  | **3** | **4** | -54.71 | 2756 | -2.02 | 0.0430 | -107.71 | -1.71 |
|  | **Severe Schizophrenia** | **1** | **2** | 168.07 | 2756 | 7.76 | <.0001 | 125.58 | 210.57 |
|  |  |  | **3** | 168.62 | 2756 | 7.78 | <.0001 | 126.12 | 211.11 |
|  |  |  | **4** | 38.24 | 2756 | 1.76 | 0.0778 | -4.25 | 80.73 |
|  |  | **2** | **3** | 0.54 | 2756 | 0.02 | 0.9801 | -41.95 | 43.03 |
|  |  |  | **4** | -129.84 | 2756 | -5.99 | <.0001 | -172.33 | -87.34 |
|  |  | **3** | **4** | -130.38 | 2756 | -6.02 | <.0001 | -172.87 | -87.89 |
| **11** | **Control** | **1** | **2** | 43.43 | 2756 | 2.40 | 0.0167 | 7.88 | 78.99 |
|  |  |  | **3** | 65.44 | 2756 | 3.61 | 0.0003 | 29.89 | 101.00 |
|  |  |  | **4** | 24.16 | 2756 | 1.33 | 0.1827 | -11.39 | 59.72 |
|  |  | **2** | **3** | 22.01 | 2756 | 1.21 | 0.2249 | -13.54 | 57.56 |
|  |  |  | **4** | -19.27 | 2756 | -1.06 | 0.2880 | -54.82 | 16.28 |
|  |  | **3** | **4** | -41.28 | 2756 | -2.28 | 0.0229 | -76.83 | -5.73 |
|  | **Mild Schizophrenia** | **1** | **2** | 72.78 | 2756 | 2.20 | 0.0280 | 7.87 | 137.69 |
|  |  |  | **3** | 62.50 | 2756 | 1.89 | 0.0591 | -2.41 | 127.41 |
|  |  |  | **4** | 11.94 | 2756 | 0.36 | 0.7183 | -52.96 | 76.85 |
|  |  | **2** | **3** | -10.28 | 2756 | -0.31 | 0.7562 | -75.19 | 54.63 |
|  |  |  | **4** | -60.83 | 2756 | -1.84 | 0.0662 | -125.74 | 4.08 |
|  |  | **3** | **4** | -50.56 | 2756 | -1.53 | 0.1268 | -115.46 | 14.35 |
|  | **Severe Schizophrenia** | **1** | **2** | 151.36 | 2756 | 6.47 | <.0001 | 105.47 | 197.26 |
|  |  |  | **3** | 148.72 | 2756 | 6.35 | <.0001 | 102.82 | 194.62 |
|  |  |  | **4** | 18.52 | 2756 | 0.79 | 0.4288 | -27.37 | 64.42 |
|  |  | **2** | **3** | -2.65 | 2756 | -0.11 | 0.9100 | -48.54 | 43.25 |
|  |  |  | **4** | -132.84 | 2756 | -5.68 | <.0001 | -178.74 | -86.94 |
|  |  | **3** | **4** | -130.19 | 2756 | -5.56 | <.0001 | -176.09 | -84.30 |
| **12** | **Control** | **1** | **2** | 71.46 | 2756 | 3.18 | 0.0015 | 27.37 | 115.56 |
|  |  |  | **3** | 63.03 | 2756 | 2.80 | 0.0051 | 18.93 | 107.12 |
|  |  |  | **4** | 50.14 | 2756 | 2.23 | 0.0259 | 6.04 | 94.24 |
|  |  | **2** | **3** | -8.44 | 2756 | -0.38 | 0.7075 | -52.54 | 35.66 |
|  |  |  | **4** | -21.32 | 2756 | -0.95 | 0.3431 | -65.42 | 22.77 |
|  |  | **3** | **4** | -12.88 | 2756 | -0.57 | 0.5667 | -56.98 | 31.21 |
|  | **Mild Schizophrenia** | **1** | **2** | 43.04 | 2756 | 0.92 | 0.3580 | -48.75 | 134.84 |
|  |  |  | **3** | 58.46 | 2756 | 1.25 | 0.2119 | -33.34 | 150.25 |
|  |  |  | **4** | 5.03 | 2756 | 0.11 | 0.9145 | -86.77 | 96.82 |
|  |  | **2** | **3** | 15.42 | 2756 | 0.33 | 0.7419 | -76.38 | 107.21 |
|  |  |  | **4** | -38.01 | 2756 | -0.81 | 0.4169 | -129.81 | 53.78 |
|  |  | **3** | **4** | -53.43 | 2756 | -1.14 | 0.2538 | -145.23 | 38.36 |
|  | **Severe Schizophrenia** | **1** | **2** | 151.50 | 2756 | 5.61 | <.0001 | 98.51 | 204.50 |
|  |  |  | **3** | 154.89 | 2756 | 5.73 | <.0001 | 101.89 | 207.89 |
|  |  |  | **4** | 17.24 | 2756 | 0.64 | 0.5237 | -35.76 | 70.23 |
|  |  | **2** | **3** | 3.38 | 2756 | 0.13 | 0.9004 | -49.61 | 56.38 |
|  |  |  | **4** | -134.27 | 2756 | -4.97 | <.0001 | -187.27 | -81.27 |
|  |  | **3** | **4** | -137.65 | 2756 | -5.09 | <.0001 | -190.65 | -84.65 |
| **13** | **Control** | **1** | **2** | 40.59 | 2756 | 1.73 | 0.0830 | -5.30 | 86.49 |
|  |  |  | **3** | 37.89 | 2756 | 1.62 | 0.1056 | -8.01 | 83.79 |
|  |  |  | **4** | 28.36 | 2756 | 1.21 | 0.2257 | -17.53 | 74.26 |
|  |  | **2** | **3** | -2.70 | 2756 | -0.12 | 0.9080 | -48.60 | 43.19 |
|  |  |  | **4** | -12.23 | 2756 | -0.52 | 0.6014 | -58.13 | 33.67 |
|  |  | **3** | **4** | -9.52 | 2756 | -0.41 | 0.6841 | -55.42 | 36.37 |
|  | **Mild Schizophrenia** | **1** | **2** | 71.07 | 2756 | 2.15 | 0.0319 | 6.16 | 135.98 |
|  |  |  | **3** | 69.51 | 2756 | 2.10 | 0.0358 | 4.60 | 134.42 |
|  |  |  | **4** | 24.74 | 2756 | 0.75 | 0.4550 | -40.17 | 89.65 |
|  |  | **2** | **3** | -1.56 | 2756 | -0.05 | 0.9624 | -66.47 | 63.35 |
|  |  |  | **4** | -46.33 | 2756 | -1.40 | 0.1617 | -111.24 | 18.58 |
|  |  | **3** | **4** | -44.77 | 2756 | -1.35 | 0.1763 | -109.68 | 20.14 |
|  | **Severe Schizophrenia** | **1** | **2** | 187.41 | 2756 | 6.93 | <.0001 | 134.41 | 240.41 |
|  |  |  | **3** | 182.41 | 2756 | 6.75 | <.0001 | 129.41 | 235.41 |
|  |  |  | **4** | 50.27 | 2756 | 1.86 | 0.0630 | -2.73 | 103.27 |
|  |  | **2** | **3** | -5.00 | 2756 | -0.18 | 0.8533 | -58.00 | 48.00 |
|  |  |  | **4** | -137.14 | 2756 | -5.07 | <.0001 | -190.14 | -84.15 |
|  |  | **3** | **4** | -132.14 | 2756 | -4.89 | <.0001 | -185.14 | -79.15 |
| **14** | **Control** | **1** | **2** | 61.80 | 2756 | 2.75 | 0.0060 | 17.71 | 105.90 |
|  |  |  | **3** | 44.03 | 2756 | 1.96 | 0.0504 | -0.07 | 88.13 |
|  |  |  | **4** | 38.24 | 2756 | 1.70 | 0.0892 | -5.86 | 82.34 |
|  |  | **2** | **3** | -17.78 | 2756 | -0.79 | 0.4294 | -61.87 | 26.32 |
|  |  |  | **4** | -23.56 | 2756 | -1.05 | 0.2948 | -67.66 | 20.53 |
|  |  | **3** | **4** | -5.79 | 2756 | -0.26 | 0.7969 | -49.89 | 38.31 |
|  | **Mild Schizophrenia** | **1** | **2** | 21.75 | 2756 | 0.66 | 0.5112 | -43.16 | 86.66 |
|  |  |  | **3** | 59.42 | 2756 | 1.80 | 0.0727 | -5.49 | 124.33 |
|  |  |  | **4** | 4.54 | 2756 | 0.14 | 0.8909 | -60.37 | 69.45 |
|  |  | **2** | **3** | 37.67 | 2756 | 1.14 | 0.2552 | -27.24 | 102.58 |
|  |  |  | **4** | -17.21 | 2756 | -0.52 | 0.6032 | -82.12 | 47.70 |
|  |  | **3** | **4** | -54.88 | 2756 | -1.66 | 0.0974 | -119.79 | 10.03 |
|  | **Severe Schizophrenia** | **1** | **2** | 152.89 | 2756 | 5.66 | <.0001 | 99.89 | 205.89 |
|  |  |  | **3** | 135.26 | 2756 | 5.00 | <.0001 | 82.26 | 188.26 |
|  |  |  | **4** | -7.57 | 2756 | -0.28 | 0.7795 | -60.57 | 45.43 |
|  |  | **2** | **3** | -17.63 | 2756 | -0.65 | 0.5143 | -70.63 | 35.37 |
|  |  |  | **4** | -160.46 | 2756 | -5.94 | <.0001 | -213.46 | -107.46 |
|  |  | **3** | **4** | -142.83 | 2756 | -5.28 | <.0001 | -195.83 | -89.83 |

Table S1 presents the per-day (up to day 14) pair wise comparisons of the LSmean (model estimated means) differences in RT between the spatial positions within each group. Note the spatial position specific effect in the different groups on different practice days. For example: a highly significant difference between the central positions and the peripheral positions on the first day of practice (CONT group) (p<<.0001) and the not significant difference between positions that share the same spatial eccentricity (p ~ 0.8).
